# Clinical Characteristics, Prognostic Factors, and Outcomes of COVID-19 in Autoimmune Rheumatic Disease Patients: A Retrospective Case–Control Study from Astana, Kazakhstan

**DOI:** 10.3390/medicina60091377

**Published:** 2024-08-23

**Authors:** Kristina Rutskaya-Moroshan, Saule Abisheva, Anilim Abisheva, Zhadra Amangeldiyeva, Tatyana Vinnik, Tansholpan Batyrkhan

**Affiliations:** 1 Department of Family Medicine №1, NJSC «Astana Medical University», Astana 010000, Kazakhstan; rutskayakristina@gmail.com (K.R.-M.); aanelim@bk.ru (A.A.); zhadra_82@mail.ru (Z.A.); tatyanavinnik15@gmail.com (T.V.); tansholpan.batyrkhan@gmail.com (T.B.); 2 Department of Molecular Biology, Ariel University, Ariel 40700, Israel

**Keywords:** SARS-CoV-2 infection, autoimmune diseases, disease course, prognostic factors, disease-modifying antirheumatic drugs

## Abstract

*Background:* Viral infections, including coronavirus disease 2019 (COVID-19), in patients with autoimmune rheumatic diseases (AIRDs) tend to present more severe disease. This study aims to investigate the clinical characteristics and risk factors for severe infection in rheumatologic patients. *Methods:* We included patients with a diagnosis of AIRD and COVID-19 infection between January 2022 and July 2023. Patients with AIRDs infected with SARS-CoV-2 were matched with control patients of the general population according to age (±5 years) and sex in a 1:1 ratio. Confirmed infection was defined if a patient had a positive polymerase chain reaction (PCR) test. The severity was divided into mild, moderate, severe, and critical according to the guidelines of the United States National Institutes of Health (NIH). *Results:* A total of 140 individuals (37 males, 103 females; mean age 56.1 ± 11.3 years) with rheumatic disease diagnosed with COVID-19 infection were enrolled in the study. AIRDs included rheumatoid arthritis (RA) (*n* = 63, 45%), ankylosing spondylitis (AS) (*n* = 35, 25%), systemic lupus erythematosus (SLE) (*n* = 26, 8.6%), and systemic sclerosis (SSc) (*n* = 16, 11.4%). The AIRDs group had more SARS-CoV-2-related dyspnea (38.6%), arthralgia (45.7%), and depression (27.1%) than the control group (*p* = 0.004). The rate of lung infiltration on radiographic examination was higher in 58 (41.4%, *p* = 0.005) patients with rheumatic diseases than in those without them. Severe SARS-CoV-2 infection was more common in the AIRDs group than in the control group (22% vs. 12%; *p* = 0.043). *Conclusions:* Patients with AIRDs experienced more symptoms of arthralgia, depression, and dyspnea. There was a trend towards an increased severity of the disease in patients with AIRDs. Patients with arterial hypertension, diabetes, chronic lung, and kidney disease, treated with corticosteroids, had a longer duration, and high activity of autoimmune disease had an increased risk of severe COVID-19.

## 1. Introduction

The COVID-19 pandemic is the result of the SARS-CoV-2 virus, which has presented significant challenges throughout the world and has affected societies and healthcare systems around the world. To date, almost 775 million cases worldwide have been surpassed, with more than seven million deaths [1]. Protecting high-risk individuals has been an essential public health focus, although identifying these high-risk groups has been somewhat complex due to the novel nature of SARS-CoV-2. In general, patients with AIRDs are more likely to be susceptible to infections [2], including SARS-CoV-2 [3]. In these cohorts, the worse outcomes of SARS-CoV-2 have been associated with immunological alterations, damage to vital organs, and the indirect impact of the drug received [4,5,6]. However, several medications used in the routine of rheumatologists, e.g., hydroxychloroquine [7], glucocorticoids (GCs) [8], IL-6 [9], and anti-TNF inhibitors [10], have been repurposed for the potential treatment of SARS-CoV-2.

During the pandemic, the practice of health care in Kazakhstan has undergone some profound difficulties. In addition to the existing risks of severe infection, patients with AIRD found themselves in a zone of “increased vulnerability”. This was explained by the inability of local health authorities to provide diagnostic investigations and procure antirheumatic drugs due to a shortage of supplies. Patients with rheumatological conditions experiencing exacerbations and those requiring regular infusions of biological antirheumatic drugs encountered significant challenges with hospitalisation and access to specialized care. These difficulties were particularly pronounced in remote areas of the country, where access to well-equipped hospitals and medical care was more limited [11]. Along with the implementation of the distance from consultation [12], the availability of national diagnostic–therapeutic disease-specific protocols (based on international guidelines even before the pandemic), contributed, to some extent, to reducing the negative consequences for individuals affected by rheumatic diseases in Kazakhstan.

In recent years, large cohort and multicentral studies have investigated how patients with AIRDs have fared with respect to COVID-19. On the contrary, there is still a paucity of research data from Central Asian countries, particularly from Kazakhstan, on the pandemic influence on rheumatology patients. Studying the clinical features and prognostic factors of SARS-CoV-2 in rheumatological patients in our region will help fill a specific gap in national research, contribute to the development of national guidelines, and support the effective clinical management of rheumatological patients during and after the pandemic. This research investigated the SARS-CoV-2 characteristics of the four most prevalent AIRDs in Kazakhstan, including RA and AS, followed by SLE and SSc [13]. The primary objective was to compare the clinical characteristics, hospitalisation rates, and outcomes of COVID-19 patients with those of healthy controls. Secondary analyses focused on determining risk factors associated with the severity of the infection.

## 2. Materials and Methods

### 2.1. Study Design

The multi-centre retrospective observational case–control study was conducted to evaluate whether patients with AIRDs and infected with COVID-19 are at a higher risk of more severe clinical manifestations than those without an AIRDs. The research was carried out in city medical clinics in the capital of Kazakhstan, Astana city, in the period from January 2022 to July 2023.

### 2.2. Settings

In Kazakhstan, the first case of COVID-19 infection was recorded in March 2020, and the cases were considerably elevated from April 2020 [14]. The first vaccine was implemented on 1 February with the Russian Gam-COVID-Vac vaccine (Sputnik V) vaccine. Since 26 April 2021 the, Kazakhstan-made QazCovid-In vaccine (QazVac) has been administered after the third phase of clinical trials [15]. During the wave of pandemics, the local health system has provided free vaccination, testing systems, and COVID-19 treatment to all residents. The public vaccine programme succeeded in vaccinating 70% of the 20 million people living in Kazakhstan by 26 November 2023, and 36% were vaccinated with at least one booster dose. The Delta variant of SARS-CoV-2 was predominant in the country after July 2021. As of March 2024, Kazakhstan had surpassed more than 1.5 million SARS-CoV-2 cases, resulting in almost 19 thousand deaths.

### 2.3. Participant Selection

Inclusion and exclusion criteria were established to determine the patient’s eligibility for the study. The inclusion criteria consisted of the following factors:

- Patients 18 years of age and older.
- Patients tested with a positive reverse transcription RNA PCR test against SARS-CoV-2.
- Patients with confirmed rheumatic diseases such as SLE, RA, AS, and SSC before SARS-CoV-2 infection. The recruited diagnoses were explained by these four most prevalent AIRDs in Kazakhstan [13].
- Patients who have filled out the information consent for the processing of personal data and participation in the survey.

The following exclusion criteria were applied:

- Patients with an unclear diagnosis purely on the basis of symptoms and other rheumatic diseases.
- Patients under the age of 18.
- Patients residing outside of Astana city. The place of residence is indicated on the title page of the case note. The study did not include patients with registered residence in other cities or regions of Kazakhstan.
- Patients who died from SARS-CoV-2.

The final sample consisted of 140 individuals with AIRDs in Astana city. We used the online sample size calculator to determine the minimum number of subjects to enrol in a study for adequate power. Gender distribution was considered irrespective of gender identity, while the age criteria set the minimum age at 18.

Control group: adult participants who did not receive immunosuppressive therapy from the general population and who were positive for the SARS-CoV-2 PCR test (*n* = 140). The control group was matched 1 to 1 with rheumatologic patients of age and sex.

### 2.4. Registration of COVID-19 Patients and Results

During the research period, we used the comprehensive patient self-reporting questionnaire for the initial recruitment of outpatient rheumatological patients. The questionnaire validation procedure consisted of the following stages: development, discussion with experts, translation, and pilot testing. The questionnaire was developed according to international standards under the direct control of the research supervisor. The questionnaire consisted of a total of 20 questions: 6 questions about personal data, 7 questions related to the clinical characteristics of AIRD, and 7 questions based on COVID-19 infection. Details on demographics, background and comorbidities, treatment and vaccination status, diagnostic tests for COVID-19, rheumatic disease activity at the beginning and after infection, symptoms, and patient-reported outcome measures of COVID-19 were also included. Demonstration of the original version of the questionnaire took place at the extended meeting of the Department of Family Medicine No1 NJSC ‘Astana Medical University’ (2021-19-10-EXP-3). At the next stage, we translated the version No. 1 into Kazakh language with the assistance of professional medical interpreters and created the pilot version of the questionnaire. For providing the free of biases and effective in collecting the intended data, the survey was tested in a pilot regime on a limited sample size (*n* = 20) in the period from November 2022 to December 2023.

The percentage of unanswered questions allowed us to evaluate how accessible the questions were for the participants to understand. In addition, the interviewees provided feedback for more precise and accurate wording of the questions. Following the pilot phase, the final version of the questionnaire was launched in January 2022. Informed consent was obtained from each patient individually before data collection.

Participants were instructed to complete the sections only if they had accurate information. Before the analysis, we contacted the patients to clarify any inaccuracies. For the mitigation of potential biases from self-reporting method, the received answers were checked manually by cross-referencing of medical records.

Additionally, we analysed patients’ electronic medical cards to gather accurate information about comorbidities, autoimmune diseases, and characteristics of COVID-19. COVID-19 characteristics included information on diagnosis, treatment, hospitalisation, and complications. Data on rheumatic diseases included clarification of diagnosis, duration, and prior AIRD activity (categorised as remission, low/moderate, severe, and unknown). At the start of COVID-19 treatment, details of GCs in prednisone equivalent doses (up to or more than 10 mg, unknown, without therapy), disease-modifying conventional synthetic antirheumatic drugs (csDMARDs) or biologic antirheumatic drugs (bDMARD) were collected. The history of comorbidities included chronic lung disease (asthma, obstructive or interstitial lung disease) and kidney disease, hypertension, cardiovascular and cerebrovascular pathology, diabetes, obesity, cancer, hepatitis, psoriasis, inflammatory bowel disease, and thyroid pathology.

We used the updated United States NIH COVID-19 treatment guidelines to categorise severity into mild, moderate, severe, or critical disease, according to clinical and radiological criteria [16]. SARS-CoV-2 reported outcomes included outpatient management, hospitalisation without oxygen requirements, hospitalisation with oxygen requirements or invasive mechanical ventilation, admission to the intensive care unit (ICU), and the development of complications.

### 2.5. The Sample Size Calculation

The main endpoint in the sample calculation was the occurrence of COVID-19-associated pneumonia and the development of severe COVID-19-associated pneumonia in patients with AIRD compared to those without AIRDS (control group). Initial study of the relevant literature revealed that severe pneumonia occurs in 14–15% of patients with COVID-19 [17,18,19]. Based on the expert opinion, we calculated that severe pneumonia would occur approximately about 2 times more often, i.e., 30%, in patients with AIRDs. Based on these figures, we calculated the minimum required sample size (Table 1).

Using the Fleiss formula [21] with continuity correction, it was determined that the minimum sample size required for each group was 134 observations. Rounding it up, we recruited 140 patients in each sample in case of dropout from the study or possible incomplete records.

### 2.6. Statistical Analysis

Rheumatological patients were matched according to age (±5 years) and sex, with SARS-CoV-2-positive results without AIRDs. Numbers and percentages were used to summarize categorical data. Continuous data were presented as medians or means ± SD, as appropriate. The Shapiro–Wilk test was employed to determine whether continuous numerical variables followed a normal distribution. Differences between categorical variables were evaluated using the chi-square test or Fisher’s exact test. For comparisons of continuous variables related to disease-specific characteristics between cases and controls, either Student’s *t*-test or the Mann–Whitney test was used, as appropriate. Binary logistic regression analysis was conducted to explore the association between the binary dependent variable (severe infection) and independent factors, using a logit model. A *p*-value < 0.05 was considered statistically significant in all tests. Statistical analyses were performed using IBM SPSS software (version 19).

### 2.7. Ethical Statement

The study received approval from the Ethics Committee of the NJSC Astana Medical University (2022-31-01-EXP-5). The study was not funded. This research was carried out in compliance with the ethical principles outlined in the Declaration of Helsinki.

## 3. Results

### 3.1. Demographic and Baseline Clinical Characteristics

During the study period, 140 patients with AIRDs and confirmed SARS-CoV-2 infection were registered. We also included 140 COVID-19-matched individuals without AIRD from the general population. Both study groups were balanced in terms of baseline characteristics, including age and sex. The average age of the patients in the case group was 56.1 years (±11.3), and in the control group, it was 51.5 years (±13.6). Both groups were predominantly female, with 73.6% (*n* = 103) in the case group and 69.3% (*n* = 97) in the control group. Smoking was observed in 16% of patients with AIRD compared to 23% in the control group.

Arterial hypertension was notably more frequent in AIRD patients than in the control group (32.1% vs. 20%, *p* = 0.021). There were no significant differences in other comorbidities. In the case group, 15.7% had lung disease, 14.3% had thyroid problems, 10% had diabetes, and 8.6% had cardiovascular disease. In the control group, 20% had hypertension, 10.7% had thyroid disease, 9.3% had lung disease, 6.4% had diabetes, and 5.7% had cardiovascular disease. Table 2 summarises the baseline demographic and clinical characteristics of the research groups.

#### 3.1.1. AIRDs Baseline Characteristics

The most prevalent rheumatic disease was RA, consisting of 63 patients (45%), followed by AS with 35 patients (25%) and SLE with 26 patients (18.6%). The SSc subgroup represented 11.4% of the cases (*n* = 16). The duration of rheumatic disease was classified as follows: 20 (14.3%) patients had a diagnosis under five years, 45 (32.1%) patients between five and nine years, and 75 (53.6%) for ten years or more. At the onset of infection, most patients (37.1%) had low or moderate disease activity, 33.6% were in remission, while 22.9% had high disease activity. The status of the disease activity was unknown in 6.4% of the individuals.

At the beginning of COVID-19, 72.1% of the patients (*n* = 101) were receiving treatment with at least one conventional DMARD. Methotrexate and leflunomide were the most commonly used Cs-DMARDs in 52 (37.1%) and 19 (13.6%) patients, followed by azathioprine (*n* = 12, 8.6%). Biological DMARDs were used in 40 patients (28.6%), with golimumab being most commonly used (*n* = 23, 16.4%). None of the patients were taking rituximab. A considerable number of patients received baseline oral glucocorticoids (*n* = 80, 42.9%). More details on the underlying diseases and immunosuppressive therapy are provided in Table 3.

#### 3.1.2. SARS-CoV-2 Infection Characteristics of the Study Groups and Results

In both groups, the majority of patients (*n* = 215, 76.8%) showed symptoms related to SARS-CoV-2, while the remaining were asymptomatic. Before SARS-CoV-2 identification, only 32 individuals with AIRDs (22.9%) had no symptoms. The most commonly reported symptoms in both groups were fever (48.9%) and headache (40.4%), followed by cough (39%) and fatigue (38.6%). The prevalence of arthralgia, shortness of breath, and depression symptoms was statistically higher in the AIRDs’ group (45.7% vs. 26.4%, *p* = 0.001; 38.6% vs. 22.9%, *p* = 0.004; 27.1% vs. 8.6%, *p* < 0.001, respectively). Headache, dysgeusia, and anosmia were higher in the control group; however, these differences did not achieve statistical significance. The presence of gastrointestinal symptoms was minimal, with vomiting and nausea observed in 7.5% of the patients. Table 4 provides a comparison of the clinical characteristics of SARS-CoV-2 infection between the study cohorts.

**Figure 1:**
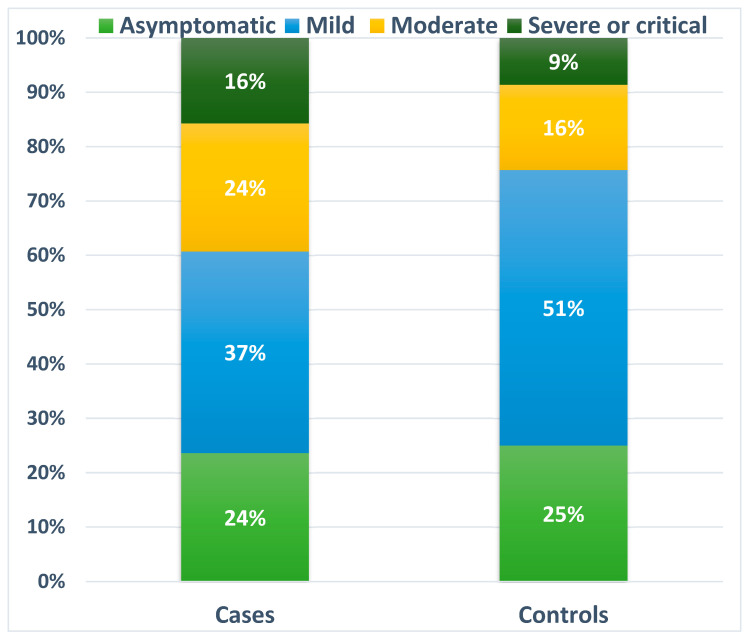
Proportions of the severity of COVID-19 in the case and control groups.

The prevalence of CT pneumonia associated with SARS-CoV-2 was significantly higher in AIRD patients (*n* = 58, 41.4%) compared to controls (*n* = 36, 25.7%, *p* = 0.05). No statistical differences were found in the CT stages of pneumonia between the study cohorts (*p* = 0.638). In the control group, 12 (8.6%) patients met the guidance criteria for severe COVID-19, while 22 patients (15.7%) with AIRDs had evidence of the severe course of infection. Other data on the severity of the disease are shown in Figure 1.

During the study period, 11 (7.8%) admissions occurred in the general population (mean age: 57.3 ± 6.6 years, 7 (63.6%) females). Among patients with AIRD, 104 (74.3%) (mean age: 48.8 ± 13.0 years, 73 (70.3%) female) did not require hospitalisation, while 36 (25.7%) (mean age: 53.9 ± 9.9 years, 30 (83.3%) female) were hospitalised. As shown, AIRDs were associated with an increased rate of hospitalisation hazard (4.1 95% CI: 2.0–8.4, *p* < 0.001). According to the prevalence of RA in the main group, the majority of the latter were patients with RA (*n* = 13, 36.1%). Among the hospitalised, 58.3% had hypertension, 41.7% had glucocorticoids at a dose of 5–10 mg daily, and 38.9% had methotrexate. Only two patients (5.6%) used bDMARDs (golimumab). Other characteristics of hospitalised patients from the case and control groups are presented in Table 5 and Table 6.

**Figure 2:**
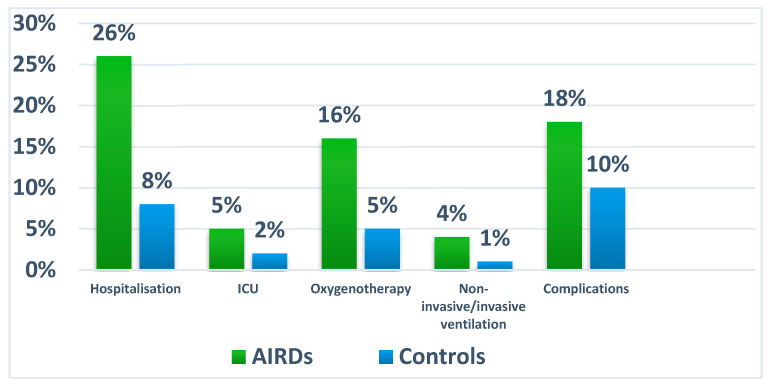
Comparison of COVID-19 outcomes between study groups.

Among patients with AIRD, 30 (83.3%) were treated on the ward, and 6 (16.7%) were treated in the intensive care unit (ICU). The percentage of patients who required oxygen therapy was significantly higher in the AIRD group compared to the control group (23 (16.4%) vs. 5 (3.5%), *p* = 0.004). Invasive mechanical ventilation was necessary for five patients (3.5%) treated in the ICU, most of whom were women (*n* = 4) and used baseline DMARDs such as methotrexate, leflunomide, azathioprine, and sulfasalazine, all of which were discontinued during the infection period. In comparison, in the control group, three patients (2%) were treated in the ICU, with two (1.4%) requiring invasive mechanical ventilation. A statistical analysis of the results comparing patients with AIRDs with controls is presented in Figure 2.

**Figure 3:**
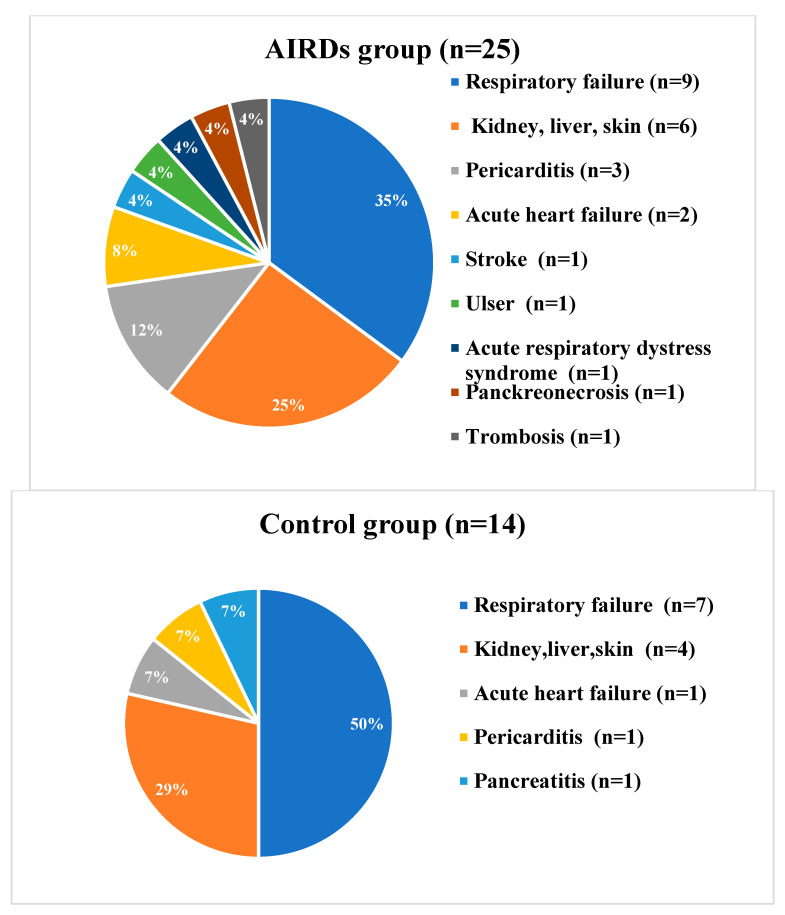
The comparison of COVID-19′s complications.

When analysing complications, 25 cases (*n* = 17.8%) were recorded in patients in the AIRD group, while only 14 people (*n* = 10%) in the control group had unfavourable consequences of infection. There were no statistically significant differences between the two groups (*p* = 0.147). As shown in Figure 2, these were respiratory failure in nine patients, pericarditis in three, acute heart failure in two, and ischemic stroke, deep vein thrombosis, ulcer, and pancreatic necrosis in one patient. Acute respiratory distress syndrome possibly associated with underlying SSc was recorded in one woman. More than two complications had five patients with AIRDs. The comparison of complications in the study groups is shown in Figure 3.

### 3.2. Factors Related to Severe SARS-CoV-2 Infection in Patients with AIRDs

We compared the main characteristics of patients with AIRD who had a mild infection with SARS-CoV-2 with those who developed a severe infection with SARS-CoV-2 in Table 7. Patients with severe SARS-CoV-2 were more likely to have the following risk factors: age over 45 years, diabetes mellitus, hypertension, cardiovascular disease, cerebrovascular disease, and chronic kidney and lung disease. It is noteworthy that 50% of patients hospitalised with AIRD who had chronic obstructive or interstitial lung disease were smokers. The high disease activity at the onset of the infection, the long duration of the disease, the GCs therapy for more than 10 years, and the dose of steroids more than 10 mg in a prednisolone equivalent were also associated with a severe course of infection.

**Figure 4:**
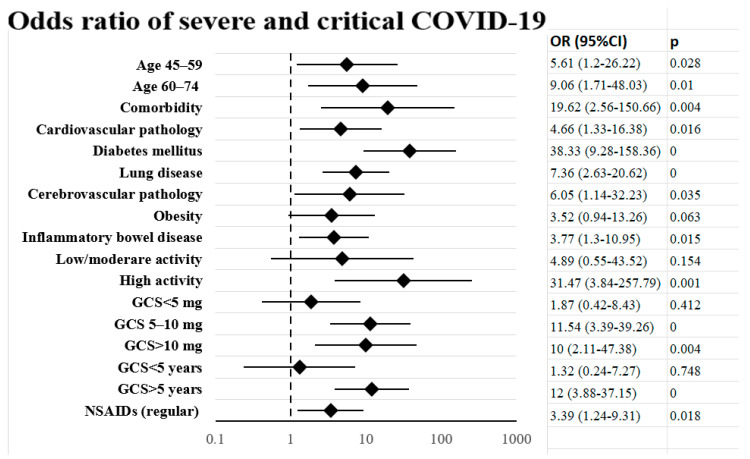
Predictive severity factors of COVID-19 in 140 patients with autoimmune rheumatic diseases.

These severity factors are also demonstrated in Figure 4, in which the *p*-values and the unadjusted odds ratios for each factor have been calculated. Although the confidence intervals were wide due to the small sample, the highest OR was found in the presence of diabetes (OR = 38.3 (9.28–158.36)) and high autoimmune activity (OR = 31.47 (3.84–257.79)).

## 4. Discussion

Patients with AIRDs have increased risks of COVID-19 infection [16,22] and severe forms of COVID-19 with mortality [23,24,25]. The “vulnerability” of this cohort can be explained for a number of reasons.

First, immune dysregulation as a result of AIRD can affect innate immune responses, which play a crucial role in preventing virus replication and developing an adaptive immune response [26]. Disability to reduce viral load in the early stages of the disease can lead to a hyperinflammatory reaction leading to tissue damage and multiple organ failure [27]. In patients with AIRD and verified COVID-19, a more severe degree of respiratory failure was observed compared to patients without a background disease [28]. Secondly, COVID-19 per se affects both the course of AIRD and antirheumatic therapeutic options. Contrary to that, the drugs used to treat AIRDs can affect the outcomes of COVID-19. Along with the studied factors of unfavourable prognosis in patients with AIRDs, the severe course of COVID-19 was associated with the use of GCS, anti-B-cell therapy [6,29], and Janus-kinase inhibitors (JAK) [30]. Thirdly, the peculiarities of the COVID-19 in patients with AIRDs echoed the issues of short-term infection or persistent neurological, respiratory, cardiovascular, endocrine consequences (including the post-COVID syndrome), or consequences of vaccination against COVID-19, with the need to study the frequency of this manifestations as well as their further impact on the course of the systemic disease. It is important to highlight that the increased exposure of rheumatological patients to COVID-19 has significant public health implications, particularly regarding their ability to work. This situation results in economic strain not only for individual families, but also on a broader social scale [31].

Our research shows that people with AIRDs had a higher susceptibility to severe SARS-CoV-2. More patients in the AIRDs group required hospitalisation, oxygen, and mechanical ventilation. These findings are consistent with those of a report on a large national cohort study in Denmark [32], in which patients diagnosed with AIRD were more likely to suffer from severe COVID-19 with a substantial increased risk of and mechanical ventilation. In a recent retrospective study conducted in Poland [33], patients with AIRDs required considerably more high-flow nasal oxygen and/or noninvasive ventilation. Interestingly, some previous studies have not identified significant differences in hospitalisation requirements [34] and admission to the ICU [35] between cohorts. However, a metanalysis showed [22] that the incidence of hospitalisation and serious clinical treatment (ICU admission and mechanical ventilation) was significantly higher in rheumatological patients.

It is crucial to clarify the clinical data and the factors that impact the prognosis of individuals with autoimmune diseases during the SARS-CoV-2 era, especially regarding the course of the disease [36]. Recent matched case–control studies have indicated that COVID-19 infection affects individuals with and without rheumatic disease in a similar way. In Nas’s research [37], rheumatologic patients had higher rates of anosmia, ageusia, dyspnoea, myalgia, arthralgia, and gastrointestinal symptoms. In our study, arthralgia, shortness of breath, and depression symptoms were statistically higher in the AIRDs group. In contrast, patients in the control group were more likely to have headaches, dysgeusia, and anosmia.

The COVID-19 Global Rheumatology Alliance (C19-GRA) established a physician-reported registry for individuals with rheumatic diseases and COVID-19 at the start of the pandemic. This registry has offered valuable insights into the outcomes of COVID-19 for those with rheumatic conditions [38]. The C19-GRA reported [29] that cardiovascular disease, combined with hypertension and chronic lung and kidney disease, were associated with higher odds of death. This may be explained by the pathogenetic characteristics of SARS-CoV-2 viruses, of which the cytotoxic effect is mediated by tropism to the ACE-2 receptor, expressed mainly in the heart, lungs and kidneys [39,40,41]. Additionally, factors related to an increased risk of hospitalisation included advanced age, high disease activity, and treatment with GCs greater than 10 mg per day [42]. In the study, we identified risk factors for severe COVID-19 infection in rheumatological patients and confirmed that older age, hypertension, diabetes, cardiovascular, chronic lung and kidney disease, and high disease activity were positively correlated with the severity of COVID-19. These results are consistent with those reported in the existing literature [29,42,43].

Pablos et al. [34] found a higher prevalence of obesity and cardiovascular disorders in people with COVID-19 and AIRD. In particular, the prevalence of comorbidity background in our cohort consisted of 44%. We found no significant disparity between the research groups in terms of incidence and type of comorbidity, except for arterial hypertension.

In line with comorbidities, age was associated with hospitalisation during the COVID-19 pandemic in further studies [22,40]. In the study Fonseca D. et al. [44], the severity of COVID-19 correlated with an age-related increasing of the titres of autoantibodies to cardiolipin, claudine, and platelet glycoprotein, which were identified as stratification markers of severe COVID-19 patients aged ≥50 years. The severe course of COVID-19 in the elderly can also be explained by the “ageing” phenomenon of the immune system with a decrease in physiological reserves [45]. Interestingly, in our study, patients with severe infection were more likely to have an age younger than 45 years. The severe course of infection was correlated with the male sex [46,47]. We did not find a statistical significance between the sex and the severity of the COVID-19 infection, which was probably due to the small sample size, and this is one of the limitations of our study.

There is an increasing amount of literature highlighting racial and ethnic health disparities related to COVID-19. Some studies have shown that racial and ethnic minorities, particularly Asians, experience worse COVID-19 outcomes compared to others [48,49]. The C19-GRA study found that Asian, African American, and Latin patients had higher odds of hospitalisation compared to White patients [50]. Additionally, racial and ethnic minority patients with rheumatic conditions tend to face a greater burden of disease [51], with higher disease activity, poorer functional status, and lower quality of life compared to White patients [52]. Unfortunately, the small sample size in our study limited the ability to examine racial predisposition to severe outcomes of COVID-19. More research is needed to understand and address the underlying causes of these disparities, especially those related to socioeconomic status and healthcare access.

Strangfeld A. et al. have shown [29] that treatment with rituximab, azathioprine, sulfasalazine, mycophenolate mofetil, cyclosporine, tacrolimus, and GCs at an equivalent dose of prednisolone of 10 mg/day was also associated with increased mortality risks compared to methotrexate monotherapy. According to data from the French cohort of rheumatological COVID-19, rheumatological drugs associated with severe infection were corticosteroids, mycophenolate mofetil, and rituximab [53]. On the contrary, b-DMARDs, such as antitumor necrosis factor α, were not associated with severe COVID-19 infection and appear to lead to a reduction in the risk of severe COVID-19 [10]. In our study, 101 patients used cs-DMARD, and 40 patients used b/DMARD. Some associations between cs-DMARDs and the severity of infection were not detected in our study. Conversely, the proportion of b/DMARD-received patients among non-hospitalised cohort was significantly higher. This circumstance allows one to consider the use of b/DMARD as a protective factor of the course of the severe infection. Previous evidence obtained from other studies showed an increased severity of the disease in patients receiving rituximab [54,55], which could lead to persistent viremia without low viral clearance [56,57]. Notably, none of the patients in our study received anti-B cell therapy.

Other unique risk factors for patients with ARDs with a possible impact on the severity of SARS-CoV-2 infection were the use of oral GCs. Corticosteroid treatment was associated with a more severe course of the disease, and the negative impact of oral corticosteroids, regardless of the indication, has been well proved in recent studies [6,29,58,59]. Treatment with higher dosages of glucocorticoids (>10 mg/day prednisolone-equivalent dose vs. no use) was also found to be associated with hospitalisation and mortality [29]. The probable reasons for this are the high activity of AIRD, which requires increasing the dose of GCS [29], as well as the potentially negative effect of steroid therapy on the process of viral replication [43]. In our rheumatic disease group, more than 42% were on baseline GCs therapy, of whom 62.5% used low doses (≤10 mg/day). As we found, GCS in a dose > 10 mg/day was significantly associated with severe SARS-CoV-2. In contrast, the efficacy of high-dose GCs for the treatment of SARS-CoV-2 was shown in the RECOVERY study [60]. The cause of this discrepancy may be explained by the timing of use in relation to the diagnosis of SARS-CoV-2 [43].

In our study, we found significant differences in terms of infection development and AIRD duration; however, no clear relationship between these aspects has been observed in the literature.

### Limitations and Strengths of the Study

Our study has some limitations that should be considered when interpreting the results, including the lack of laboratory parameters, such as C-reactive protein, ferritin, and interleukins levels. In this retrospective design, data collection could be subject to potential biases and affect all groups of patients, both cases and controls, in the same way. In particular, there was a rapid development of scientific knowledge around COVID-19, including the development of COVID-19 vaccines and the mutation of virus variants. Unfortunately, our data were collected over a long period of time and may not reflect the current situation. Interpreting the prevalence of depression symptoms in the main group of our study could introduce bias, as these symptoms are generally more common in individuals with AIRDs than in the general population. Additionally, a significant limitation of our research was the exclusion of deceased COVID-19 patients from the outcome measures, which could lead to bias, particularly in underestimating the impact of COVID-19 on the AIRD population in our region. This exclusion may also affect the results by hindering a comprehensive understanding of the full spectrum of disease severity. Lastly, the sample size was insufficient to include a broader range of diagnoses, limiting our ability to extrapolate the effects of rare specific diagnoses or less commonly used DMARDs on COVID-19 infection.

However, our study has several strengths. We manually recruited the patient sample evaluated within multiple medical centres in a single city. Subsequently, using the online DAMUMED database, we were able to conduct a case–control study matched with propensity score, adjusted for age, sex, and comorbidities commonly associated with the severity of COVID-19. These factors were well balanced between the two groups. Free access to patient medical records allowed us to verify information on the diagnosis of underlying AIRDs, comorbidities, treatment, and outcomes. The study population was from a single centre with a unified management protocol for COVID-19 infection, which eliminated inconsistencies in quality of care between study subjects. The included outcomes measures for oxygen therapy and ventilation support were objective guided by objective clinical measures and were not affected by caregiver decisions or pandemic circumstances. On the contrary, due to the limitations of resources during pandemic peaks, hospitalisation criteria could change, affecting study outcomes. Our definition of severe SARS-CoV-2 infection was compatible with the WHO definition and recommended in the updated WHO guidelines for September 2020. All SARS-CoV-2 cases were confirmed by PCR tests in medical centres, which were providers of COVID-19 care in Astana during the pandemic. Importantly, the extensive tracking and testing strategy applied in Kazakhstan allowed us to identify cases with mild or no symptoms. Therefore, our study reflects the true rate of severe COVID-19 infection. Finally, to our knowledge, this is the first study in our region that has provided global data on the prognostic factors of this infection in individuals with rheumatic diseases.

## 5. Conclusions

In this study, we extracted data from Astana, Kazakhstan, on the baseline characteristics, outcomes, and risk factors of COVID-19 in patients with AIRDs. Compared to non-AIRD controls, AIRD patients required hospitalisation, oxygen therapy, and invasive mechanical ventilation more frequently. The age over 45 years, diabetes mellitus, hypertension, cardiovascular and cerebrovascular disease, and chronic kidney and chronic lung disease were identified as risk factors for the severe SARS-CoV-2. The AIRD-mediated factors, as the high disease activity, the duration of the disease, the GCs therapy for more than 10 years, and the dose more than 10 mg in a prednisolone equivalent were also associated with a severe course of infection.

The authors hope that the results contribute to the growing volume of evidence on the clinical characteristics and outcomes of SARS-CoV-2 in this cohort of patients. In summary, our study had serious limitations of the small sample size, retrospective design, potential biases in terms of using self-report data, and exclusion of COVID-19-associated death from the outcome measures. Therefore, more research with larger sample sizes is needed to further characterise the infection process in patients with AIRDs. Realising regional guidelines and research can help accumulate personal experience and knowledge on aspects of SARS-CoV-2 and rheumatology, as well as optimising the treatment of rheumatological patients in the post-pandemic era.

## Figures and Tables

**Table 1:** The sample size calculation for the case–control study.

For:			
	Two-sided confidence level (1-alpha)	95	
	Power (% chance of detecting)	80	
	Ratio of Controls to Cases	1	
	Hypothetical proportion of controls with exposure	15	
	Hypothetical proportion of cases with exposure:	30	
	Least extreme Odds Ratio to be detected:	2.43	
	Kelsey [20]	Fleiss [21]	Fleiss with CC
Sample Size-Cases	122	121	134
Sample Size-Controls	122	121	134
Total sample size:	244	242	268
CC = continuity correction			
Results are rounded up to the nearest integer.			
Results from OpenEpi, Version 3, open-source calculator—SSCC			

**Table 2:** The primary demographic and clinical characteristics of the research groups.

	AIRDS	Control Group
SEX n (%)		
Female	103 (73.6%)	97 (69.3%)
Male	37 (26.4%)	43 (30.7%)
Age, mean (SD)	56.1 ± 11.3	51.5 ± 13.6
18–44	46	50
45–59	64	69
60–74	24	19
75–90	6	2
Smoker	22	32
Comorbidities		
Hypertension	45 (32.1%)	28 (20%)
Diabetes	14 (10%)	9 (6.4%)
Cardiovascular disease	12 (8.6)	8 (5.7%)
Cerebrovascular disease	6 (4.3%)	4 (2.9%)
Chronic lung disease	22 (15.7%)	13 (9.3%)
Chronic kidney disease	4 (2.9%)	2 (1.4%)
Cancer	5 (3.6%)	3 (2.1%)
Psoriasis	3 (2.1%)	6 (4.3%)
Hepatitis	4 (2.9%)	3 (2.1%)
Inflammatory bowel disease	10 (7.1%)	4 (2.9%)
Hypo/hyperthyroidism	20 (14.3%)	15 (10.7%)
Nationality		
Kazakh	103	100
Russian	22	18
Tatars	6	7
Ukrainian	3	4
Poles	3	6
Germans	2	5
Greeks	1	0

**Table 3:** Details on immunosuppressive therapy for underlying diseases.

Therapy	Diagnosis							
	RA		AS		SSc		SLE	
No NSAIDs (regular)	50	79.4%	24	68.6%	6	100.0%	25	96.2%
NSAIDs (regular)	13	20.6%	11	31.4%	0	0.0%	1	3.8%
No csDMARDs therapy	15	23.8%	14	40.0%	7	43.8%	3	11.5%
Metotrexate	31	49.2%	17	48.6%	4	25.0%	0	0.0%
Leflunomide	10	15.9%	4	11.4%	3	18.8%	2	7.7%
Mycophenolatmophetil	0	0.0%	0	0.0%	0	0.0%	7	26.9%
Sulfasalazine	0	0.0%	1	6.3%	0	0.0%	0	0.0%
Hydroxychloroquine	7	11.1%	0	0.0%	0	0.0%	3	11.5%
Azathioprine	0	0.0%	0	0.0%	1	6.3%	11	42.3%
No steroids	40	63.5%	35	100.0%	2	12.5%	3	11.5%
Up to 5 mg	12	19.0%	0	0.0%	8	50.0%	7	26.9%
5–10 mg	11	17.5%	0	0.0%	6	37.5%	6	23.1%
More than 10 mg	0	0.0%	0	0.0%	0	0.0%	10	38.5%
No bDMARDs therapy	48	76.2%	10	28.6%	16	100.0%	26	100.0%
Golimumab	9	14.3%	14	40.0%	0	0.0%	0	0.0%
Adalimumab	1	1.6%	6	17.1%	0	0.0%	0	0.0%
Tocilizumab	5	7.9%	2	5.7%	0	0.0%	0	0.0%
Infliximab	0	0.0%	3	8.6%	0	0.0%	0	0.0%

Notes: NSAIDs: non-steroidal anti-inflammatory drugs, csDMARD: conventional synthetic disease-modifying antirheumatic drug, bDMARDs: biologic disease-modifying antirheumatic drugs.

**Table 4:** Clinical features of SARS-CoV-2 infection in the study groups.

Indicators		Study Group				*p*
		Case		Controls		
		N	%	N	%	
SARS-CoV-2 pneumonia	No	82	58.6%	104	74.3%	0.006
	Yes	58	41.4%	36	25.7%	
CT-stages	>25%	20	33.9%	16	45.7%	0.681
	25–50%	22	37.3%	10	28.6%	
	50–75%	14	23.7%	7	20.0%	
	>75%	2	3.4%	2	5.7%	
Fever	No	79	56.4%	64	45.7%	0.073
	Yes	61	43.6%	76	54.3%	
Cough	No	79	56.4%	92	65.7%	0.111
	Yes	61	43.6%	48	34.3%	
Headache	No	84	60.0%	83	59.3%	0.903
	Yes	56	40.0%	57	40.7%	
Dyspnoea	No	86	61.4%	108	77.1%	0.004
	Yes	54	38.6%	32	22.9%	
Throat pain	No	102	72.9%	110	78.6%	0.265
	Yes	38	27.1%	30	21.4%	
Arthralgia	No	76	54.3%	103	73.6%	0.001
	Yes	64	45.7%	37	26.4%	
Myalgia	No	89	63.6%	91	65.0%	0.803
	Yes	51	36.4%	49	35.0%	
Dysgeusia	No	105	75.0%	96	68.6%	0.232
	Yes	35	25.0%	44	31.4%	
Anosmia	No	92	65.7%	83	59.3%	0.267
	Yes	48	34.3%	57	40.7%	
Irritability/Depression	No	102	72.9%	128	91.4%	<0.001
	Yes	38	27.1%	12	8.6%	
Asthenia/Fatigue	No	79	56.4%	93	66.4%	0.086
	Yes	61	43.6%	47	33.6%	
Diarrhoea/vomiting	No	130	92.9%	129	92.1%	0.821
	Yes	10	7.1%	11	7.9%	
Thorax pain	No	118	84.9%	120	85.7%	0.846
	Yes	21	15.1%	20	14.3%	

**Table 5:** Characteristics of hospitalised patients with AIRDs.

Patients with AIRDs		Hospitalisation				*p*
		Yes		No		
		N	%	N	%	
Gender	Male	6	16.7%	31	29.8%	0.123
	Female	30	83.3%	73	70.2%	
Age	18–44	5	13.9%	41	39.4%	0.007
	45–59	21	58.3%	43	41.3%	
	60–74	10	27.8%	14	13.5%	
	75–90	0	0.0%	6	5.8%	
Diagnosis	RA	13	36.1%	50	48.1%	0.042
	AS	6	16.7%	29	27.9%	
	SLE	9	25.0%	17	16.3%	
	SSc	8	22.2%	8	7.7%	
Duration of the disease	<5 years	4	11.1%	16	15.4%	0.153
	5–10 years	7	19.4%	38	36.5%	
	11–20 years	20	55.6%	42	40.4%	
	>20 years	5	13.9%	8	7.7%	
Comorbidity	No	5	13.9%	53	51.0%	<0.001
	Yes	31	86.1%	51	49.0%	
Smoking	No	27	75.0%	75	72.1%	0.793
	Yes	6	16.7%	16	15.4%	
	Past smokers	3	8.3%	13	12.5%	
GCS (doses)	No	7	19.4%	73	70.2%	<0.001
	<5 mg	9	25.0%	18	17.3%	
	5–10 mg	15	41.7%	8	7.7%	
	>10 mg	5	13.9%	5	4.8%	
GCs therapy duration	<5 years	9	25.0%	16	15.4%	<0.001
	5–10 years	13	36.1%	14	13.5%	
	>10 years	7	19.4%	0	0.0%	
NSAIDs (regular)	No	28	77.8%	87	83.7%	0.428
	Yes	8	22.2%	17	16.3%	
csDMARDs	No immunosuppressive therapy	9	25.0%	30	28.8%	0.492
	Metotrexate	14	38.9%	38	36.5%	
	Leflunomide	3	8.3%	16	15.4%	
	Mycophenolatmophetil	2	5.6%	5	4.8%	
	Sulfasalazine	0	0.0%	1	1.0%	
	Hydroxychloroquine	2	5.6%	8	7.7%	
	Azathioprine	6	16.7%	6	5.8%	
duration csDMARDs duration	<5 years	10	27.8%	36	34.6%	0.119
	5–10 years	10	27.8%	37	35.6%	
	>10 years	15	41.7%	27	26.0%	
bDMARDs	No biological therapy	34	94.4%	66	63.5%	0.011
	Golimumab	2	5.6%	21	20.2%	
	Adalimumab	0	0.0%	7	6.7%	
	Tocilizumab	0	0.0%	7	6.7%	
	Infliximab	0	0.0%	3	2.9%	

Notes: RA: rheumatoid arthritis; AS: ankylosing spondylitis; SLE: systemic lupus erythematosus; SSc: systemic sclerosis; GCS: glucocorticosteroids; NSAIDs non-steroidal anti-inflammatory drugs; csDMARDs: conventional synthetic disease-modifying antirheumatic drugs; bDMARDs: biologic disease-modifying antirheumatic drugs.

**Table 6:** Characteristics of hospitalised patients (control group).

Control Group		Hospitalisation				*p*
		Yes		No		
		N	%	N	%	
Gender	Male	4	36.4%	43	33.3%	0.838
	Female	7	63.6%	86	66.7%	
Age	18–44	0	0.0%	86	66.7%	<0.001
	45–59	1	9.1%	34	26.4%	
	60–74	8	72.7%	9	7.0%	
	75–90	2	18.2%	0	0.0%	
Comorbidity	No	0	0.0%	86	66.7%	<0.001
	Yes	11	100.0%	43	33.3%	
Smoking	No	6	54.5%	85	65.9%	0.719
	Yes	3	27.3%	29	22.5%	
	Past smokers	2	18.2%	15	11.6%	

**Table 7:** Factors associated with severe and non-severe SARS-CoV-2 infection in 140 patients with autoimmune rheumatic diseases.

Factors		SARS-CoV-2 Severity				*p*
		Mild/Moderate		Severe/Critical		
		n	%	n	%	
Gender	Male	33	28.0%	4	18.2%	0.339
	Female	85	72.0%	18	81.8%	
Age	18–44	44	37.3%	2	9.1%	0.019
	45–59	51	43.2%	13	59.1%	
	60–74	17	14.4%	7	31.8%	
	75–90	6	5.1%	0	0.0%	
Diagnosis	RA	53	44.9%	10	45.5%	0.840
	AS	31	26.3%	4	18.2%	
	SSc	13	11.0%	3	13.6%	
	*SLE*	21	17.8%	5	22.7%	
Comorbidity		61	51.7%	21	95.5%	0.000
Hypertension		28	23.7%	17	77.3%	0.000
Cardiovascular pathology		7	5.9%	5	22.7%	0.010
Diabetes mellitus		3	2.5%	11	50.0%	0.000
Chronic obstructive/interstitial lung disease		12	10.2%	10	45.5%	0.000
Cerebrovascular pathology		3	2.5%	3	13.6%	0.018
Cancer		3	2.5%	2	9.1%	0.129
Obesity		7	5.9%	4	18.2%	0.050
Psoriasis		3	2.5%	0	.0%	0.450
Hepatitis		3	2.5%	1	4.5%	0.605
Chronic kidney disease		0	0.0%	4	18.2%	0.000
Hypo/hyperthyroidism		7	5.9%	3	13.6%	0.198
Inflammatory bowel disease		13	11.0%	7	31.8%	0.010
Smoking	No	85	72.0%	17	77.3%	0.144
	Yes	17	14.4%	5	22.7%	
	Past smokers	16	13.6%	0	0%	
Disease activity at the time of infection	Remission	46	39.0%	1	4.5%	0.000
	Low/moderate	47	39.8%	5	22.7%	
	High activity	19	16.1%	13	59.1%	
	Unknown	6	5.1%	3	13.6%	
GCS (doses)	No	75	63.6%	5	22.7%	0.000
	<5 mg	24	20.3%	3	13.6%	
	5–10 mg	13	11.0%	10	45.5%	
	>10 mg	6	5.1%	4	18.2%	
AIRD duration	<5 years	18	15.3%	2	9.1%	0.011
	5–10 years	43	36.4%	2	9.1%	
	11–20 years	49	41.5%	13	59.1%	
	>20 years	8	6.8%	5	22.7%	
GCS therapy duration	No	76	64.4%	5	22.7%	0.000
	1–5 years	23	19.5%	2	9.1%	
	<5 years	19	16.1%	15	68.2%	
Duration csDMARDs	5–10 years	45	38.1%	6	27.3%	0.082
	>10 years	42	35.6%	5	22.7%	
	>10 years	31	26.3%	11	50.0%	
csDMARDs	No immunosuppressive therapy	34	28.8%	5	22.7%	0.955
	Metotrexate	43	36.4%	9	40.9%	
	Leflunomide	16	13.6%	3	13.6%	
	Mycophenolatmophetil	6	5.1%	1	4.5%	
	Sulfasalazine	1	0.8%	0	0.0%	
	Hydroxychloroquine	9	7.6%	1	4.5%	
	Azathioprine	9	7.6%	3	13.6%	
NSAIDs (regular)		17	14.4%	8	36.4%	0.014
bDMARDs	No biological therapy	79	66.9%	21	95.5%	0.111
	Golimumab	22	18.6%	1	4.5%	
	Adalimumab	7	5.9%	0	0.0%	
	Tocilizumab	7	5.9%	0	0.0%	
	Infliximab	3	2.5%	0	0.0%	

Notes: GCS: glucocorticosteroids; NSAIDs: non-steroidal anti-inflammatory drugs; csDMARDs: conventional synthetic disease-modifying antirheumatic drugs; bDMARDs: biologic disease-modifying antirheumatic drugs.

## Data Availability

The original contributions presented in the study are included in the article, further inquiries can be directed to the corresponding authors.
